# Efficacy of perioperative chemotherapy for synovial sarcoma: a retrospective analysis of a Nationwide database in Japan

**DOI:** 10.1186/s12885-021-08485-1

**Published:** 2021-07-03

**Authors:** Gang Xu, Hisaki Aiba, Norio Yamamoto, Katsuhiro Hayashi, Akihiko Takeuchi, Shinji Miwa, Takashi Higuchi, Kensaku Abe, Yuta Taniguchi, Yoshihiro Araki, Shiro Saito, Kenichi Yoshimura, Hideki Murakami, Hiroyuki Tsuchiya, Akira Kawai

**Affiliations:** 1 Department of Orthopaedic Surgery, Kanazawa University Graduate School of Medical Sciences, Kanazawa, Japan; 2 Department of Musculoskeletal Tumor, First Affiliated Hospital of Shenzhen University, Second People’s Hospital, Shenzhen, China; 3 Department of Orthopedic Surgery, Nagoya City University Graduate School of Medical Sciences, Nagoya, Japan; 4 Department of Biostatistics, Medical Center for Translational and Clinical Research, Hiroshima University Hospital, Hiroshima, Japan; 5 Department of Musculoskeletal Oncology and Rehabilitation, National Cancer Center Hospital, 5-1-1 Tsukiji, Chuo-ku, Tokyo, 104-0045 Japan

**Keywords:** Perioperative chemotherapy, Chemotherapy, Soft-tissue sarcoma, Synovial sarcoma, Database study, Matched-pair analysis

## Abstract

**Background:**

Synovial sarcoma is an aggressive but chemosensitive soft-tissue tumor. We retrospectively analyzed the efficacy of perioperative chemotherapy for synovial sarcoma with data from the nationwide database, Bone and Soft Tissue Tumor Registry in Japan.

**Methods:**

This study included 316 patients diagnosed with synovial sarcoma between 2006 and 2012. Oncologic outcomes were analyzed using a Cox-hazard regression model. Moreover, the effects of perioperative chemotherapy on outcomes were evaluated using a matched-pair analysis. The oncologic outcomes of patients who did or did not receive chemotherapy were compared (cx + and cx-).

**Results:**

Multivariate analysis revealed significant correlations of age (over 40, hazard ratio [HR] = 0.61, *p* = 0.043), margin status (marginal resection, HR = 0.18, *p* < 0.001 and intralesional resection, HR = 0.30, *p* = 0.013 versus wide resection) with overall survival; surgical margin type (marginal resection, HR = 0.14, *p* = 0.001 and intralesional resection, HR = 0.09, *p* = 0.035 versus wide resection) with local recurrence; and postoperative local recurrence (HR = 0.30, *p* = 0.027) and surgical margin (marginal resection, HR = 0.31, *p* = 0.023 versus wide resection) with distant relapse-free survival.

Before propensity score matching, perioperative chemotherapy was mainly administered for young patients and patients with deeper tumor locations, larger tumors, more advanced-stage disease, and trunk location. The 3-year overall survival, local control, and distant relapse-free survival rates were 79.8%/89.3% (HR = 0.64, *p* = 0.114), 89.6%/93.0% (HR = 0.37, *p* = 0.171) and 71.4%/84.5% (HR = 0.60, *p* = 0.089) in the cx+/cx- groups, respectively. After propensity score matching, 152 patients were selected such that the patient demographics were nearly identical in both groups. The 3-year overall survival, local control, and distant relapse-free survival rates were 71.5%/86.0% (HR = 0.48, *p* = 0.055), 92.5%/93.3% (HR = 0.51, *p* = 0.436) and 68.4%/83.9% (HR = 0.47, *p* = 0.046) in the cx+/cx- groups, respectively.

**Conclusion:**

This large-sample study indicated that the margin status and postoperative disease control were associated directly or indirectly with improved oncologic outcomes. However, the efficacy of perioperative chemotherapy for survival outcomes in synovial sarcoma patients was not proven in this Japanese database analysis.

**Supplementary Information:**

The online version contains supplementary material available at 10.1186/s12885-021-08485-1.

## Background

Synovial sarcoma (SS), an aggressive mesenchymal tumor with high rates of local recurrence and metastasis, accounts for 5–10% of soft-tissue sarcomas (STSs). SS occurs most frequently in adolescents and young adults [1–6]. These tumors can be divided into three histologic subtypes: monophasic tumors, which are composed of spindle cells; biphasic tumors, which are composed of spindle and epithelial cells; and poorly differentiated tumors, which are composed of small round cells [4]. SS is considered to be chemosensitive [4, 7], and a wide excision with a negative margin is necessary for effective treatment [8–10]. Therefore, the administration of perioperative chemotherapy might be a rational approach to reduce micro-invasion from the primary site. However, chemotherapy for SS remains controversial because it is difficult to conduct a prospective study on the efficacy of perioperative therapy, specifically for this tumor type. Moreover, several pretreatment characteristics, including the tumor size, age, histologic grade, and tumor depth [6, 9, 11–14], influence the prognosis of a patient with SS and may have affected the results of previous studies. This study was conducted to evaluate the several prognostic factors that might affect the oncologic outcomes and to clarify the role of perioperative chemotherapy in the prognosis of SS patients based on a matched-pair analysis (MPA).

## Methods

### Patient selection

We extracted patient data from the Bone and Soft Tissue Tumor (BSTT) Registry of Japan, a nationwide organ-specific cancer registry for bone and soft-tissue tumors. Eighty-nine Japanese Orthopedic Association (JOA)-certified hospitals that specialize in musculoskeletal oncology participated obligatorily in this registry, and other hospitals participated voluntarily. The annual reports published by the BSTT include patient characteristics, such as basic data (sex, age, date of diagnosis, and treatment status at first visit [history of treatment in previous hospitals], tumor data (diagnosis, histologic details [malignant or benign disease and the histologic grade for malignant tumors]), tumor location, data required for TNM staging (American Joint Committee on Cancer staging system, 7th edition), surgical data (date of definitive surgery, type of surgery, reconstruction details, and additional surgeries for complications), and information about additional treatments (chemotherapy, radiotherapy, and hyperthermia) [15]. Follow-up surveys were conducted to collect information after 2, 5, and 10 years following the initial registration. These surveys included outcomes, such as local recurrence, distant metastasis, and oncologic outcomes, at the time of the latest follow-up. This study was approved by the Institutional Review Board of the JOA.

**Fig. 1:**
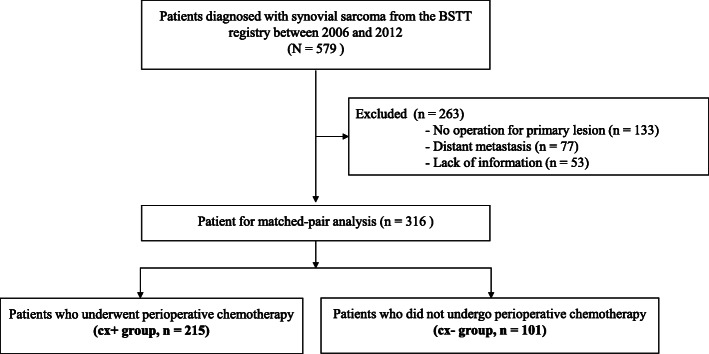
A CONSORT diagram of patient selection for this study

From the BSTT registry, we identified 579 patients who were diagnosed with SS between 2006 and 2012. Of these, we excluded 133 patients who did not undergo primary tumor resection, 53 patients with missing data, and 77 patients with metastatic lesions. The final analysis dataset included 316 patients (Fig. 1).

### Statistical analysis

The primary objective of this study was to investigate the following oncologic outcomes: overall survival (OS), defined as the time from diagnosis to death from any cause; distant relapse-free survival (D-RFS), defined as the time from surgery to distant progression or death; and local control (LC), defined as the time from surgery to local recurrence. Standardized intergroup differences were calculated using Kaplan–Meier and log-rank analyses. Potential risk factors for oncologic outcomes were analyzed with a step-wise Cox proportional hazards model, and hazard ratios (HRs) were calculated from these data.

We divided patients into two groups based on treatment with or without perioperative chemotherapy (cx + versus cx- group). For the MPA, data on statistical variables, including age; sex; tumor location, size, stage, histology, depth, and margin status; and adjuvant radiotherapy, were obtained from the BSTT registry. A multivariate logistic regression analysis was conducted to determine associations between these factors and the administration of perioperative chemotherapy. Propensity scores were calculated using a logistic regression model that included the weights of the contributions of each patient’s demographic data. After calculating these scores, we propensity score-matched patients in a 1:1 ratio by using a nearest-neighbor algorithm, allowing a maximum tolerated difference of ≤30% between propensity scores [16]. In addition, to evaluate the high-risk population, we performed a similar analysis separately for stage III patients (*n* = 147) and repeated MPA as per the abovementioned methods. All statistical analyses were conducted using SPSS version 25 (IBM Corp., Armonk, NY, USA). A two-sided *p*-value < 0.05 was considered statistically significant.

## Results

This analysis included 316 patients (age, mean ± SD, 38.8 ± 18.2; males, 153 [48.3%]; females, 163 [51.7%]). The median follow-up period (interquartile range) was 939 (473–1279) days. The tumors were located in the lower extremities, upper extremities, trunk, and head/neck in 160 (50.5%), 56 (17.7%), 93 (29.3%), and 7 (2.2%) patients, respectively. Furthermore, the tumor subtypes were monophasic, biphasic, and unclassified/undifferentiated in 153 (48.3%), 89 (28.2%), and 74 (23.4%) patients, respectively. The median (interquartile range) tumor size was 5.4 (3.4–9.0) cm. The tumor stages were Ia-b, IIa-b, and III in 12 (3.8%), 157 (49.5%), and 147 (46.4%) patients, respectively.

### Oncologic outcomes

#### Overall survival

The included patients had OS rates of 83.5% (±2.6) at 3 years and 66.8% (±4.5) at 5 years. In the univariate analysis, sex, tumor subtype, tumor depth, tumor size, and tumor location had no significant impacts on OS. However, there were significant correlations of age (over 40, hazard ratio [HR] = 0.56, *p* = 0.019). Also, the surgical margin type (marginal resection, HR = 0.16, *p* < 0.001; intralesional resection, HR = 0.29, *p* = 0.016 versus wide resection) and administration of postoperative radiotherapy (HR = 0.58, *p* = 0.018) were associated significantly with a poorer outcome. The age and surgical margins were retained in the multivariate analysis (Table 1).

#### Local control

The postoperative local recurrence rates were 9.1% (±2.1) at 3 years and 11.1% (±2.9%) at 5 years. In the univariate analysis, sex, tumor subtype, tumor depth, tumor size, and tumor location had no significant effects on LC. Moreover, the surgical margin type (marginal resection, HR = 0.12, *p* = 0.011; intralesional resection, HR = 0.08, *p* = 0.022, compared with wide resection) and administration of radiotherapy (HR = 0.24, *p* = 0.001) were significantly associated with local recurrence. Surgical margins were retained in the multivariate analysis (Table 1).

#### Distant relapse-free survival

Patients in our sample had D-RFS rates of 80.2% (±2.7) at 3 years and 68.7% (±4.5) at 5 years. In the univariate analysis, sex, tumor subtype, tumor depth, tumor size, and tumor location had no significant effects on D-RFS. However, significant associations with age (over 40, hazard ratio [HR] = 0.56, *p* = 0.016), postoperative local recurrence (HR = 033, *p* = 0.004), inadequate surgical margin (marginal resection, HR = 0.21, *p* < 0.01, compared with wide resection), and administration of radiotherapy (HR = 0.44, *p* = 0.02) were identified. Surgical margins and local recurrence remained significant in the multivariate analysis (Table 1).

**Table 1:** Results of univariate and multivariate analyses of oncologic outcomes

Characteristics	Number	HR (95% CI)	***P***-value	HR (95% CI)	***P***-value
*Overall survival*		*Univariate analysis*		*Multivariate analysis*	
Sex (male < female)	153/163	0.70 (0.42–1.08)	0.104		
Age (Over 40 < Under 40)	141/175	0.56 (0.35–0.91)	0.019	0.61 (0.38–0.99)	0.043
FNCLCC grade (3 < 2)	304/12	0.68 (0.09–4.93)	0.704		
Subtype (Unclassified < Biphasic)	74/89	0.57 (0.27–1.21)	0.143		
Subtype (Monophasic < Biphasic)	153/89	0.82 (0.45–1.51)	0.529		
Depth (Superficial < Deep)	45/271	0.59 (0.24–1.48)	0.263		
Tumor size (Under 5 cm < Over 5 cm)	117/199	0.73 (0.42–1.29)	0.277		
Tumor location (Upper < Lower extremity)	56/160	0.81 (0.47–1.41)	0.811		
Tumor location (Trunk+head and neck < Lower extremity)	100/216	0.81 (0.39–1.68)	0.813		
Surgical margin (Marginal < Wide)	35/268	0.16 (0.07–0.36)	< 0.01	0.18 (0.08–0.38)	< 0.01
Surgical margin (Intralesional < Wide)	13/268	0.29 (0.11–0.79)	0.016	0.30 (0.11–0.78)	0.013
Adjuvant radiotherapy (Yes < No)	56/260	0.58 (0.32–0.90)	0.018		
Perioperative chemotherapy (Yes < No)	215/101	0.64 (0.35–1.14)	0.114		
Neoadjuvant (Yes < No)	165/151	0.73 (0.28–1.88)	0.508		
Adjuvant (Yes < No)	131/185	0.48 (0.16–1.42)	0.182		
*Local control*		*Univariate analysis*		*Multivariate analysis*	
Sex (male < female)	153/163	0.94 (0.42–2.49)	0.940		
Age (Over 40 < Under 40)	141/175	0.95 (0.39–2.32)	0.910		
FNCLCC grade (3 < 2))	304/12	0.05^a^	0.636		
Subtype (Unclassified < Biphasic)	74/89	0.58 (0.17–2.70)	0.580		
Subtype (Monophasic < Biphasic)	153/89	0.82 (0.32–3.12)	0.990		
Depth (Superficial < Deep)	45/271	0.33 (0.04–2.49)	0.284		
Tumor size (Under 5 cm < Over 5 cm)	117/199	0.61 (0.22–1.70)	0.340		
Tumor location (Upper < Lower extremity)	56/160	0.76 (0.28–2.05)	0.592		
Tumor location (Trunk+head and neck < Lower extremity)	100/216	0.67 (0.17–2.58)	0.557		
Surgical margin (Marginal < Wide)	35/268	0.12 (0.04–0.36)	0.011	0.14 (0.05–0.44)	0.001
Surgical margin (Intralesional < Wide)	13/268	0.08 (0.01–0.70)	0.022	0.09 (0.01–0.84)	0.035
Adjuvant radiotherapy (Yes < No)	56/260	0.24 (0.10–0.58)	0.001		
Perioperative chemotherapy (Yes < No)	215/101	0.37 (0.12–1.12)	0.171		
Neoadjuvant (Yes < No)	165/151	0.97 (0.40–2.32)	0.936		
Adjuvant (Yes < No)	131/185	0.55 (0.20–1.51)	0.248		
*Distal recurrent survival*		*Univariate analysis*		*Multivariate analysis*	
Sex (male < female)	153/163	0.60 (0.36–1.01)	0.053	NS	
Age (Over 40 < Under 40)	141/175	0.56 (0.35–0.90)	0.016	0.63 (0.39–1.03)	0.06
FNCLCC grade (3 < 2))	304/12	0.43 (0.06–3.13)	0.407		
Subtype (Unclassified < Biphasic)	74/89	0.57 (0.27–1.24)	0.160		
Subtype (Monophasic < Biphasic)	153/89	0.86 (0.46–1.61)	0.640		
Depth (Superficial < Deep)	45/271	0.53 (0.21–1.31	0.284		
Tumor size (Under 5 cm < Over 5 cm)	117/199	0.62 (0.35–1.11)	0.340		
Tumor location (Upper < Lower extremity)	56/160	0.80 (0.45–2.05)	0.592		
Tumor location (Trunk+head and neck < Lower extremity)	100/216	0.81 (0.39–1.70)	0.557		
Postoperative local recurrence (Yes < No)	69/247	0.33 (0.16–0.71)	0.004	0.30 (0.17–0.90)	0.027
Surgical margin (Marginal < Wide)	35/268	0.21 (0.08–0.54)	0.001	0.31 (0.11–0.85)	0.023
Surgical margin (Intralesional < Wide)	13/268	0.46 (0.15–1.38)	0.105	0.71 (0.21–2.34)	0.571
Adjuvant radiotherapy (Yes < No)	56/260	0.44 (0.26–0.73)	0.002		
Perioperative chemotherapy (Yes < No)	215/101	0.60(0.33–1.08)	0.089		
Neoadjuvant (Yes < No)	165/151	0.86(0.54–1.40)	0.562		
Adjuvant (Yes < No)	131/185	0.62(0.37–1.05)	0.073		

*CI* confidence interval, *FNCLCC* Fédération Nationale des Centres de Lutte Contre le Cancer

^a^95% CI was scaled out

#### Contribution of chemotherapy to oncologic outcomes

**Fig. 2:**
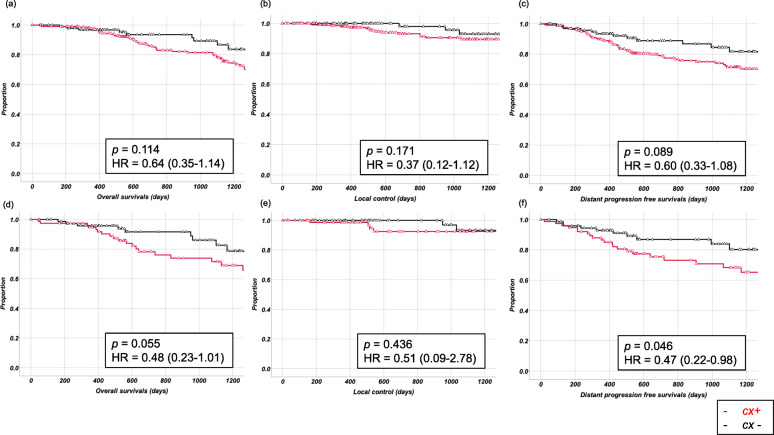
Kaplan-Meier analyses of oncologic outcomes. The oncologic outcomes of patients who did (cx+) or did not (cx-) receive chemotherapy were compared (red curve: cx + group, black curve: cx- group). **a**–**c** outcomes before propensity-score matching (*n* = 316); **d**–**f** outcomes after propensity-score matching (*n* = 152). Triangles indicate the censored cases. **a** The overall survival of patients with/without chemotherapy before propensity-score matching. **b** The local control rate of patients with/without chemotherapy before propensity-score matching. **c** The distant progression-free survival of patients with/without chemotherapy before propensity-score matching. **d** The overall survival of patients with/without chemotherapy after propensity-score matching. **e** The local control rate of patients with/without chemotherapy after propensity-score matching. **f** The distant progression-free survival of patients with/without chemotherapy after propensity-score matching

Before adjustment with the propensity score, we observed some differences between patients who did (*n* = 215) or did not (*n* = 101) receive perioperative chemotherapy; particularly, the former group tended to be younger and to have deeper tumor locations, larger tumors, more advanced-stage disease, and monophasic-type disease compared to the latter group (Table 2). In the cx + group, most patients underwent perioperative chemotherapy with either the adriamycin + ifosfamide (AI) regimen or another doxorubicin regimen, administered along with cisplatin, ifosfamide, dacarbazine, or vincristine (Table 3). The 3-year OS rates were 79.8% (±3.3%) in the cx + group and 89.3% (±4.0%) in the cx- group (HR = 0.64 [0.35–1.14], *p* = 0.114), and the 3-year LC rates were 89.6% (±2.6%) in the cx + group and 93.0% (±3.9%) in the cx- group (HR = 0.37 [0.12–1.12], *p* = 0.171). The 3-year D-RFS rates were 71.4% (±3.6%) in the cx + group and 84.5% (±5.2%) in the cx- group (HR = 0.60 [0.33–1.08], *p* = 0.089; Fig. 2).

**Table 2:** Patient characteristics before and after the matched-pair analysis

		Before matching (***N*** = 316)			After matching (***N*** = 152)		
		Chemotherapy (***n*** = 215)	No chemotherapy (***n*** = 101)	***P***-value	Chemotherapy (***n*** = 76)	No chemotherapy (***n*** = 76)	***P***-value
Sex	Male/female	108/107	45/56	0.345^a^	38/38	34/42	0.516^a^
Age, years	< 20/20–40/40–60/> 60 Mean, SD	39/101/54/21 34.7, 16.3	11/24/39/27 47.2, 19.1	< 0.001 ^b^	15/21/26/16 41.3, 18.5	8/23/26/19 45.9, 19.2	0.797^b^
FNCLCC grade	Grade 2/grade 3	4/211	8/93	0.009	2/74	4/72	0.405
Depth	Superficial/deep	20/195	25/76	< 0.001^a^	13/63	14/62	0.882^a^
Location	Trunk/head and neck/upper extremity/lower extremity	71/7/37/100	22/0/19/60	0.035^a^	17/0/19/40	8/0/17/41	0.927^a^
Length of tumor, cm	< 5/5–10/10–15/> 15 Median, IQR	65/96/46/8 6.8, 3.6	52/33/8/8 5.4, 3.9	< 0.001^b^	35/29/8/4 6.1, 3.9	36/28/8/4 5.4, 3.5	0.999^b^
Stage	I/II/III	4/94/117	8/63/30	0.07^a^	2/45/29	1/48/27	0.882^a^
Subtype	Monophasic/biphasic/unclassified	102/63/50	51/26/24	0.799	39/17/20	38/19/19	0.985^a^
Surgical Margin	Wide/marginal/intralesional	185/20/10	83/15/3	0.286	62/12/2	64/9/3	0.719
Adjuvant radiotherapy	Yes/no	41/174	15/86	0.360	4/72	3/73	0.699

*SD* standard deviation, *FNCLCC* Fédération Nationale des Centres de Lutte Contre le Cancer, *IQR* interquartile range

^a^Chi-square test, ^b^Mann-Whitney *U* test

**Table 3:** Chemotherapy regimens

Drug agents	Numbers (***n*** = 215)
A + I	132
A + I + E + P	10
I	9
A + I + D	9
A + I + E	8
A + I + P	6
I + E	4
Other A containing regimens	37

*A* adriamycin, *D* dacarbazine, *E* etoposide, *I* ifosfamide, *P* cisplatin

After propensity score matching, 152 patients were selected such that the patient demographics were nearly identical in both the groups (Table 2). The 3-year OS rates were 71.5% (±6.0%) in the cx + group and 86.0% (±5.1%) in the cx- group (HR = 0.48 [0.23–1.01], *p* = 0.055), and the 3-year LC rates were 92.5% (±3.7%) in the cx + group and 93.3% (± 4.0%) in the cx- group (HR = 0.51 [0.09–2.78], *p* = 0.436). The 3-year D-RFS rates were 68.4% (±6.2%) in the cx + group and 83.9% (±5.2%) in the cx- group (HR = 0.47 [0.22–0.98], *p* = 0.046, Fig. 2).

#### Analysis of oncologic outcomes of the extracted subgroup consisting of stage III patients

For a preliminary analysis, 147 high-risk stage III patients were exclusively extracted into a subgroup Their baseline characteristics are shown in Additional file 1. In total, 117 patients underwent chemotherapy with a predilection for young patients with large tumors. Before matching, there was no improvement in the oncologic outcomes with perioperative chemotherapy. After matching, 52 cases were almost identical in the two groups. However, there was still no improvement in the oncologic outcomes (Additional file 2).

## Discussion

Definitive treatment strategies for SS have not been fully determined. Moreover, its associated risk factors, including distant metastasis at diagnosis, SS subtype, tumor depth, and tumor size, influence the oncologic outcomes and makes it difficult to obtain meaningful results [6, 7, 9, 12–14]. Therefore, we analyzed data from the largest soft-tissue tumor-specific database in Japan to determine the risk factors associated with SS outcomes.

We identified that surgical margins and local recurrence after primary resection affected the oncologic outcomes. These findings indicated the importance of complete surgical resection to avoid micro/macro-residues of the tumor in the post-resection margins. Chemotherapy could potentially reduce possible invasion around the tumor and thus prevent micro-residual resection. In cases where a tumor arises near neurovascular bundles, neoadjuvant chemotherapy improves the likelihood of sparing the neurovascular bundles during resection and enabling the patient to forgo amputation, which allows the preservation of muscle function [17]. Furthermore, perioperative chemotherapy can potentially improve patient survival by eradicating micro-metastatic disease.

Currently, the role of preoperative chemotherapy in SS remains controversial [18] because of the challenges associated with prospective studies and the potential for various selection biases in retrospective studies. A meta-analysis of 14 trials reported that doxorubicin-based chemotherapy significantly improved oncologic outcomes. The SS subgroup extracted from these trials was better oriented for chemotherapy. Nonetheless, the analysis identified no significant improvement in OS (57.5 and 47.3% for the chemotherapy and control groups, respectively) [19]. Eilber et al. [5] reported favorable outcomes with ifosfamide-based chemotherapy for SS in a dataset limited to patients with tumors > 5 cm, deep tumors, as well as primary and extremity tumors who were treated between 1990 and 2002. In that study, the 4-year disease-specific survival rates were 88 and 67% in the chemotherapy and no-chemotherapy groups, respectively (*p* = 0.01). Additionally, treatment with an ifosfamide-based regimen was reported to improve D-RFS (HR = 0.4, *p* = 0.03) [13]. Ferrari et al. [20] suggested that patients aged ≥17 years and those with tumors > 5 cm achieved better outcomes with chemotherapy. Based on these results, several prospective trials in Europe (European Paediatric Soft Tissue Sarcoma Study Group [21]) and the United States (Children’s Oncology Group [22]) reported risk-adapted perioperative treatment with AI chemotherapy and radiotherapy. In these studies, low-risk patients (with completely resected tumor < 5 cm in size) were treated with surgery alone. Corresponding to these results, in Japan, the JOA recommended not to administer chemotherapy for low-risk patients, especially for young patients [23]. Based on the results of the nationwide study JCOG 0304, the standard schedule is 3 cycles of neoadjuvant and 2 cycles of adjuvant therapy with AI [24]. However, the indications for chemotherapy were not yet standardized in Japan.

In contrast, few published reports have focused on neoadjuvant therapy for the treatment of SS and reported no clinical benefit on outcomes [25]. One randomized phase 2 trial of adult patients with high-risk STS (tumor size > 8 cm of any grade, tumor size < 8 cm of grade 2/3, or locally recurrent sarcoma/after inadequate surgery of grade 2/3) indicated that a regimen of 3 cycles of neoadjuvant chemotherapy was not superior to surgery alone in the included patients (5-year disease-free survival rates of 56 and 52% for the neoadjuvant chemotherapy and surgery-alone arms, respectively; *p* = 0.354) [26]. Similarly, localized SS in an Italian study group were treated with a combination of ifosfamide and doxorubicin or epirubicin, and the 5-year OS rates of those who did or did not receive chemotherapy were 69 and 82%, respectively (*p* = 0.20). In that study, the negative impact of chemotherapy was explained by the exclusive administration of this treatment modality to patients with larger tumors (> 5 cm) and those with re-excision. These preconditions may have influenced the outcomes [9]. However, that study did not sufficiently balance the number of patients who received neoadjuvant chemotherapy with those who received adjuvant therapy. Therefore, it remains difficult to draw meaningful conclusions on the actual contribution of neoadjuvant chemotherapy to patient outcomes.

Preoperative or postoperative radiotherapy is now widely administered for stage II or III soft tissue tumors. Preoperative chemoradiotherapy is a treatment option validated by the prospective study of the Radiation Therapy Oncology Group (RTOG9514 [27]) and database retrospective study on MPA [28]. In Japan, the administration of radiotherapy is recommended only for cases with inadequate margins in adjuvant settings [23]. In this study, adjuvant radiotherapy was mainly administered to the patients with inadequate margins (29/268 patients with wide margins and 27/48 with marginal or intralesional margins). Thus, selection bias may have a negative effect on oncologic outcomes in patients receiving adjuvant radiotherapy.

To reduce the possible bias of the retrospective analysis, we examined the oncologic outcomes of SS using an MPA of a relatively large population and, thus, have presented a novel report. Before MPA, we found that the cx + population had larger tumors, deeper locations, younger age, and axial locations, suggesting that selection bias might affect the oncologic outcomes. Despite propensity matching to reduce intergroup differences, we did not observe significant differences in the oncologic outcomes of patients in the cx + and cx- groups. This result might be criticized because the MPA acted towards reducing high-risk patients in the cx + group (e.g., 117 stage III patients in the cx + group were reduced to 29 patients after adjustments). To address this issue, we re-analyzed the stage III patients separately; however, we were not able to indicate the superiority of neoadjuvant over adjuvant chemotherapy even in the extracted group consisting of stage III patients. Recently, a similar methodological study that used the National Cancer Database reported improved OS with chemotherapy in the MPA of stage III soft tissue sarcoma patients, especially in the undifferentiated pleomorphic sarcoma group [29]. This conflicting tendency was also suggested by major referral centers in Europe [30]. These trends may be attributed to the fact that Japanese clinicians exclusively administer chemotherapy for complicated cases based on their own clinical judgement, which was not quantified in the BSTT database.

This study had several limitations. First, the design was retrospective, and therefore, many biases, including selection and recall bias, may have influenced the results despite the propensity-score adjustment. Unmeasured confounders, which were not incorporated into the database, might have affected the result of this study. Second, the BSTT database consists only of patients treated at orthopedic departments; thus, our dataset did not include patients treated at other departments (e.g., retroperitoneal tumors treated in the urology department). Third, the quality of the database may affect the results, including the relatively short observation periods, the lack of detailed information on the exact schedule and intensity of chemotherapy, the accuracy of the diagnosis or histological grade, and details regarding the surgical procedure. Fourth, we did not analyze differences in genotypes. Fusion proteins resulting from *SYT-SSY1* or *SYT-SSY2* fusions have been associated with the histological subtype and clinical behavior. In addition, the CINSARC signature has been accepted as the prognostic evaluation based on the mitosis and chromosome integrity; therefore, these biomarkers should be reviewed in a further analysis of this database [31, 32]. Finally, in Japan, the standard treatment protocol for SS involves a doxorubicin-based chemotherapy regimen. However, the different participating institutions do not use identical protocols. Accordingly, although 61.3% of patients received neoadjuvant chemotherapy via the AI regimen, many patients were treated with AI-ifosfamide + etoposide, A + cisplatin, mesna + A + I + dacarbazine, or other regimens. These differences might have affected the study outcomes. We further note that, currently, new drugs are being approved rapidly in Japan, and pazopanib, trabectedin, and eribulin have been proven to yield improved oncologic outcomes in patients. These newly approved drugs may influence patient outcomes and, therefore, potential changes in treatment strategy should be considered when applying our findings.

## Conclusions

We analyzed a large population database in Japan to determine the factors that affect the oncologic outcomes of patients with non-metastatic SS. Notably, we found that the margin status and postoperative local control were associated directly or indirectly with improvements in oncologic outcomes. However, we did not find a significant contribution of perioperative chemotherapy to survival outcomes in either the non-adjusted or propensity score-matched populations.

## Supplementary Information

**Additional file 1.** The characteristics of stage III patients before and after MPA.

**Additional file 2 **The Kaplan-Meier curves of stage III patients before and after MPA. Kaplan-Meier analyses of oncologic outcomes (extracted stage III patients). The oncologic outcomes of patients who did (cx+) or did not (cx-) receive neoadjuvant chemotherapy were compared (red curve: cx + group, black curve: cx- group). a–c: outcomes before propensity-score matching (*n* = 147); d–f: outcomes after propensity-score matching (*n* = 52). Triangles indicate the censored cases. (a) The overall survival of patients with/without chemotherapy before propensity-score matching. (b) The local control rate of patients with/without chemotherapy before propensity-score matching. (c) The distant progression-free survival of patients with/without chemotherapy before propensity-score matching. (d) The overall survival of patients with/without chemotherapy after propensity-score matching. (e) The local control rate of patients with/without chemotherapy after propensity-score matching. (f) The distant progression-free survival of patients with/without chemotherapy after propensity-score matching.

## Data Availability

The datasets that support the findings of this study are available on request from the Japanese Orthopedic Association committee.

## Abbreviations

AI: Adriamycin + ifosfamide
BSTT: Bone and Soft Tissue Tumor
cx: Neoadjuvant chemotherapy
D-RFS: Distant relapse-free survival
HR: Hazard ratio
JOA: Japanese Orthopedic Association
LC: Local control
MPA: Matched-pair analysis
OS: Overall survival
STS: Soft-tissue sarcomas
SS: Synovial sarcoma
