# Supplementary material for: Efficacy of perioperative chemotherapy for synovial sarcoma: a retrospective analysis of a Nationwide database in Japan
Source: BMC Cancer. 2021 Jul 3;21:773. doi: 10.1186/s12885-021-08485-1 (PMC8255009; doi:10.1186/s12885-021-08485-1)
Supplement: Supplementary file 1 — Additional file 1. The characteristics of stage III patients before and after MPA. [file 12885_2021_8485_MOESM1_ESM.docx]

**Additional Table 1**. The characteristics of stage III patients before and after the matched-pair analysis

|  |  | **Before matching (N = 147)** | | | **After matching (N = 52)** | | |
| --- | --- | --- | --- | --- | --- | --- | --- |
|  |  | **Chemotherapy**  **(n = 117)** | **No chemotherapy**  **(n = 30)** | ***P*-value** | **Chemotherapy**  **(n = 26)** | **No chemotherapy**  **(n = 26)** | ***P*-value** |
| Sex | Male/female | 59/58 | 15/15 | 0.967^a^ | 13/13 | 13/13 | 1.0 |
| Age, years | <20/20–40/40–60/>60  Mean, SD | 21/56/31/9  34.3, 16.0 | 0/6/15/9  54.3, 15.2 | <0.001 ^b^ | 0/4/18/4  49.2, 12.9 | 0/6/13/7  53.4, 14.6 | 0.363^b^ |
| Location | Trunk/head and neck/upper extremity/lower extremity | 71/7/37/100 | 22/0/19/60 | 0.581 | 10/0/2/14 | 9/0/1/16 | 0.771^a^ |
| Length of tumor, cm | <5/5–10/10–15/>15  Median, IQR | 0/66/43/8  9.7, 4.0 | 0/15/8/7  9.2, 2.9 | 0.027 | 0/15/8/3  10.1, 2.9 | 0/13/7/6  9.6, 3.8 | 0.546 ^b^ |
| Subtype | Monophasic/biphasic/unclassified | 55/32/30 | 13/7/10 | 0.700 | 15/6/5 | 13/6/7 | 0.909^a^ |
| Surgical Margin | Wide/marginal/intralesional | 99/13/5 | 24/5/1 | 0.700 | 22/4/0 | 20/5/1 | 0.547 |
| Adjuvant radiotherapy | Yes/no | 20/97 | 4/26 | 0.619 | 5/21 | 3/23 | 0.442 |

^a^chi-square test, ^b^Mann–Whitney *U* test

SD: standard deviation; IQR: interquartile range
