# Supplementary figures and images for: Efficacy of perioperative chemotherapy for synovial sarcoma: a retrospective analysis of a Nationwide database in Japan
Source: BMC Cancer. 2021 Jul 3;21:773. doi: 10.1186/s12885-021-08485-1 (PMC8255009; doi:10.1186/s12885-021-08485-1)

## Slide 1
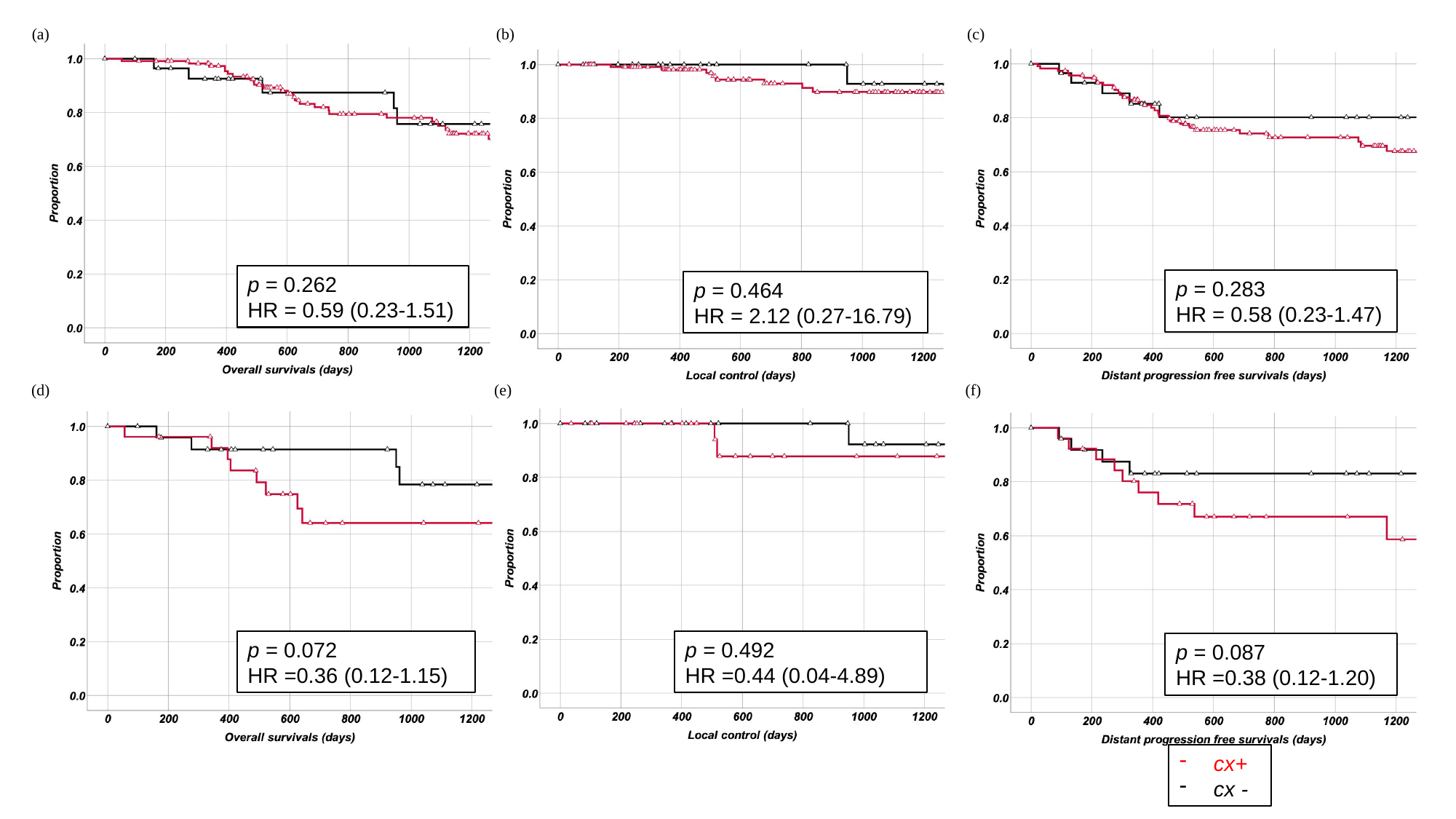

(a)
(b)
(c)
p = 0.262
HR = 0.59 (0.23-1.51)
p = 0.283
HR = 0.58 (0.23-1.47)
p = 0.464
HR = 2.12 (0.27-16.79)
(d)
(e)
(f)
p = 0.072
HR =0.36 (0.12-1.15)
p = 0.492
HR =0.44 (0.04-4.89)
p = 0.087
HR =0.38 (0.12-1.20)
cx+
cx -

Supplement: Supplementary file 2 — Additional file 2 The Kaplan-Meier curves of stage III patients before and after MPA. Kaplan-Meier analyses of oncologic outcomes (extracted stage III patients). The oncologic outcomes of patients who did (cx+) or did not (cx-) receive neoadjuvant chemotherapy were compared (red curve: cx + group, black curve: cx- group). a–c: outcomes before propensity-score matching (n = 147); d–f: outcomes after propensity-score matching (n = 52). Triangles indicate the censored cases. (a) The overall survival of patients with/without chemotherapy before propensity-score matching. (b) The local control rate of patients with/without chemotherapy before propensity-score matching. (c) The distant progression-free survival of patients with/without chemotherapy before propensity-score matching. (d) The overall survival of patients with/without chemotherapy after propensity-score matching. (e) The local control rate of patients with/without chemotherapy after propensity-score matching. (f) The distant progression-free survival of patients with/without chemotherapy after propensity-score matching. [file 12885_2021_8485_MOESM2_ESM.pptx]
